# Neuronal Calcium Signaling in Metabolic Regulation and Adaptation to Nutrient Stress

**DOI:** 10.3389/fncir.2018.00025

**Published:** 2018-04-05

**Authors:** Siddharth Jayakumar, Gaiti Hasan

**Affiliations:** National Centre for Biological Sciences, Tata Institute of Fundamental Research, Bangalore, India

**Keywords:** neuronal control of metabolism, glutamatergic neurons, hypothalamus, mNSC, AgRP neurons, *Drosophila melanogaster*

## Abstract

All organisms can respond physiologically and behaviorally to environmental fluxes in nutrient levels. Different nutrient sensing pathways exist for specific metabolites, and their inputs ultimately define appropriate nutrient uptake and metabolic homeostasis. Nutrient sensing mechanisms at the cellular level require pathways such as insulin and target of rapamycin (TOR) signaling that integrates information from different organ systems like the fat body and the gut. Such integration is essential for coordinating growth with development. Here we review the role of a newly identified set of integrative interneurons and the role of intracellular calcium signaling within these neurons, in regulating nutrient sensing under conditions of nutrient stress. A comparison of the identified *Drosophila* circuit and cellular mechanisms employed in this circuit, with vertebrate systems, suggests that the identified cell signaling mechanisms may be conserved for neural circuit function related to nutrient sensing by central neurons. The ideas proposed are potentially relevant for understanding the molecular basis of metabolic disorders, because these are frequently linked to nutritional stress.

## Introduction

The ability of an organism to sense nutrients in its environment is closely linked to survival (Lam, 2010). Nutrients are essential for all adult physiological processes and are also required for normal growth during development. To survive and adapt to an ever-changing environment, organisms need to couple nutrient sensing mechanisms to signaling pathways that allow survival through conditions of nutrient stress. Such mechanisms regulate allocation of available nutrients for essential and non-essential processes, thus modulating growth and development. This regulation is dependent on consumption of appropriate levels of food, and different organisms have evolved nutrient sensing mechanisms for optimal food consumption (Gleason et al., 2007; Miyamoto et al., 2013; Chantranupong et al., 2015). Modern human settlements evolved from simple hunter gatherers, where food availability was sporadic and thus the emphasis was on consumption of available resources at the earliest and storage of excess food as reserves for survival until the next meal (Berbesque et al., 2014). A change in lifestyle has led to assured food supply with continuous access to calorically dense food, for which human metabolism appears poorly adapted. This is very likely one of the reasons for the observed increase in diseases related to obesity, such as diabetes and cardiac dysfunction over the past 50 years (Speakman, 2013). At a cellular level, various signaling pathways exist for sensing different nutrients to ensure optimal growth (Andersen et al., 2013; Chantranupong et al., 2015). The Target of Rapamycin (TOR) and Insulin/IGF-1 signaling (IIS) pathways are well studied and are known to be conserved broadly across various phyla (Efeyan et al., 2015). Thus, an understanding of nutrient sensing mechanisms across phyla would provide insights into the regulation of metabolic decisions, specifically of those related to survival under nutrient stress.

## External Nutrient Sensors

Nutrients are macromolecules required for catabolic and anabolic processes in the cell. Organisms have evolved varying strategies to sense nutrients from their external environment, as their individual nutritional requirements maybe different (Efeyan et al., 2015). From prokaryotic organisms to multicellular eukaryotes, sensors have diversified depending on the substrate to be sensed and the environmental context of the organism. Unicellular organisms possess dedicated sensors that allow them to sense nutrients directly as they are exposed as individuals to fluxes in nutrient levels. In multicellular organisms also there are dedicated sensors for nutrients that form the bulk of nutritional content like sugars, amino acids and fatty acids (Chantranupong et al., 2015). Despite dedicated sensory neurons for different classes of nutrients (Mishra et al., 2013; Efeyan et al., 2015), direct sensing of nutrients by central neurons has also been observed (Bjordal et al., 2014; Chen et al., 2015). Therefore, there exist diverse modes of nutrient sensing in multicellular organisms with physiological implications that ensure appropriate growth and ultimately survival. Such systems and the strategies implemented to sense external nutrients by central neurons need to be studied as they could help identify phylogenetically conserved unifying principles used for context specific survival by multicellular organisms.

## Neuronal Regulation of Nutrient Sensing and Metabolic Regulation

**Figure 1:**
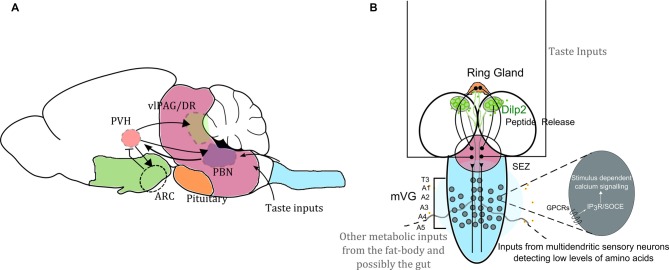
Neuronal circuit motifs regulating feeding and satiety are conserved between mammals and *Drosophila*. Comparison of circuits regulating feeding and satiety in mammals **(A)** and *Drosophila* larvae **(B)** equates the hypothalamus with the medial neurosecretory cells (mNSC; green), the pituitary region to the ring gland (orange), the brain stem to the sub-oesophageal ganglion (SOG or the sub-oesophageal zone, SEZ; pink) and the spinal cord to the ventral ganglion (blue). Studies have shown that the agouti-related peptide (AgRP) neurons and pro-opiomelanocortin (POMC) neurons housed in the arcuate nucleus (ARC) are critical for driving feeding behavior. However, in the absence of these two key cell types, feeding and satiety are also influenced by the paraventricular nucleus (PVH of the hypothalamus), which activates the cells in the ARC as well as drives activity in the para-brachial nucleus (PBN) for attaining satiety (Wu et al., 2009, 2012; Sternson and Eiselt, 2017). Glutamatergic interneurons in the mid-ventral ganglion (mVG) similarly activate the mNSC to regulate response to starvation (Jayakumar et al., 2016). In **(A)** the arrowheads show activation, and the arrow with the flatline indicates inhibition. The various regions are important for achieving satiety. In **(B)** we also highlight that intracellular calcium signaling mechanisms through various G-protein coupled receptors (GPCRs) involving both the inositol-1,4,5-trisphosphate receptor (IP_3_R) mediated calcium release as well as store-operated calcium entry (SOCE) in the mVG neurons possibly allow integration of environmental dietary quality with internal metabolic state. Hence the with internal metabolic state. Hence the mVG-mNSC connection in *Drosophila* larvae may be a precursor of an evolved and complex spinal.

Maintenance of carbohydrate homeostasis in metazoans is thought to occur predominantly through the IIS pathway whereas the maintenance of amino acid homeostasis occurs through a combination of signaling mechanisms which include TOR, General Control Non-depressible 2 kinase (GCN2) and the Adenosine 5′-Monophosphate-activated Protein Kinase (AMPK) pathways (Hardie, 2014; Chantranupong et al., 2015). Together these pathways regulate energy homeostasis. Neuronal circuits that regulate metabolic state are unique as they combine multisensory inputs and give rise to complex behavioral or developmental outputs rather than a simple motor output command (Yarmolinsky et al., 2009). They are also tightly regulated as their failure invariably affects survival. Elegant experiments in the rodent hypothalamic arcuate nucleus (ARC) and parabrachial nucleus (PBN) have shown that peptidergic neurons in the hypothalamus marked by the Agouti-related peptide (AgRP) and Pro-opiomelanocortin (POMC) regulate feeding and satiety (Sternson and Eiselt, 2017). Hunger states in rodents are also under the influence of the insular cortex, in turn regulated by AgRP neurons (Livneh et al., 2017). Similar circuits exist in *Drosophila* (Figure 1) where molecular signatures of development and anatomical similarities have led the medial Neurosecretory Cells (mNSCs) to be compared to the hypothalamus in many instances (De Velasco et al., 2004; de Velasco et al., 2007; Hartenstein, 2006). The mNSCs regulate feeding carbohydrate metabolism and other physiological processes (Broughton et al., 2005; Bader et al., 2007; Nässel and Winther, 2010; Waterson et al., 2014). These cells, exist in the developing larval brain of *Drosophila*, and thus present an excellent system to study neuronal regulation of metabolic processes during development. Unlike the mNSCs, hypothalamic circuits do not exist during vertebrate development (Waterson and Horvath, 2015). Brain regions that provide inputs to the mNSCs, as well as mNSC output centers are thought to functionally correspond to other regions of the vertebrate brain (Figure 1). Based on studies comparing the mammalian neuropeptide neuromedin-U and its *Drosophila* homolog Hugin, the brain stem in mammals is often compared to the sub-oesophageal zone (SEZ) in *Drosophila* (Nakazato et al., 2000; Melcher et al., 2006; Bader et al., 2007; Schlegel et al., 2016). The brain stem hosts the PBN as well as the dorsal raphe (DR; Peyron et al., 1997). These regions are known to regulate satiety but remain under modulation by inputs from the paraventricular hypothalamic nucleus (Sternson, 2013).

Till recently, neuronal control of metabolic state has been considered secondary in *Drosophila*, where the fat-body and endocrine tissues like the corpora cardiaca in the ring gland, along with the mid-gut are considered as the predominant tissue systems for metabolic regulation (Arrese and Soulages, 2010; Andersen et al., 2013; Rajan and Perrimon, 2013). *Drosophila* also use hormonal systems such as insulin, leptin and glucagon thus demonstrating remarkable similarities of endocrine regulation of metabolic homeostasis with vertebrates (Rulifson et al., 2002; Rajan and Perrimon, 2013; Rorsman and Braun, 2013). Additionally, recent work in mammals and flies indicate the gut-brain axis as relevant for neuroendocrine regulation to achieve metabolic homeostasis (Dus et al., 2015; Zeng et al., 2015).

In this context, we have recently shown that a class of glutamatergic interneurons in the larval mid-ventral ganglion (mVG), regulate the integration of external nutrient information with appropriate developmental transitions (Jayakumar et al., 2016). The mVG neurons exert their influence on systemic metabolism by regulating peptide release from the mNSCs (Jayakumar et al., 2016). This newly identified neural circuit appears similar to the mammalian para ventricular hypothalamus, which regulates nutritional state by regulating the ARC (Figure 1; Sternson and Eiselt, 2017).

## Signaling Mechanisms for Nutrient Sensing

Cellular mechanisms for sensing nutrients have evolved and vary across unicellular to multicellular organisms. Such sensing mechanisms often employ a range of signaling pathways like the IIS, TOR, GCN2 and AMPK in a context dependent manner (Chantranupong et al., 2015). High levels of amino acids in the environment lead to increased TOR activity (Kim and Guan, 2011) whereas high glucose levels activate the IIS pathway for growth (Polak and Hall, 2009). Similarly, available nutrient levels alter AMPK signaling as AMPK is directly activated by low ATP:ADP ratio and therefore acts as an intracellular energy sensor (Chotechuang et al., 2009). High amino acid levels suppress GCN2 which inhibits translation by phosphorylation of the eukaryotic initiation factor 2α (eIF2α; Dever and Hinnebusch, 2005; Towle, 2007). TOR and IIS also converge to regulate 4E-BP an inhibitor of the eIF4E (Jackson et al., 2010; Erion and Sehgal, 2013). Therefore, information from multiple cellular signaling pathways is important for a cell or an organism to respond and adapt to nutritional changes in the environment.

For studying signaling mechanisms that modulate nutrient sensing, a holometabolous insect like *Drosophila* is particularly useful because of the two distinct stages where they actively consume nutrients and that differ in the environmental conditions they encounter. One is the adult fly that feeds, predominantly on rotting fruits. Studies have shown that there are specific neural circuits very similar to mammals that regulate hunger and feeding in adult *Drosophila* (Nässel and Winther, 2010; Yang et al., 2011). An interesting example of this is peptidergic modulation of hunger. Thus the regulation of hunger and satiety by neuropeptide (F in insects and Y in mammals) and insulin signaling axes are conserved in insect and mammalian systems, though peptidergic signaling is more complex in vertebrates (Brogiolo et al., 2001; Wu et al., 2003; Nässel and Winther, 2010). However, because neurotransmitter identities in neuronal classes are not conserved between vertebrates and invertebrates, it is often difficult to compare insect neuronal circuits with vertebrate circuits directly.

The nutritional requirements, of *Drosophila* larval stages are also of interest because larvae undergo an approximately 200-fold increase in body size from the first to the third larval instar (Mirth et al., 2009; Grewal, 2012). Before pupariation, third instar larvae feed voraciously and accumulate sufficient nutrients and energy to tide over the stationary pupal phase. In addition, third instar larvae possess check-points to ensure that they do not pupariate before sufficient nutrients have been stored. One such check-point is the attainment of critical weight (McBrayer et al., 2007), defined as the weight beyond which absolute starvation still yields 50% pupariation (Mirth et al., 2009). The mechanism that allows *Drosophila* larvae to pupariate post critical weight even in a nutrient deficient environment has been explored recently (Jayakumar et al., 2016). Absence of external nutrient inputs are sensed by a neural circuit that signals to specific receptors on larval brain neurons where intracellular calcium signals are generated and transmitted to the mNSCs for regulating neuropeptide release. These neuropeptides, which include but are not restricted to an insulin-like peptide, help generate ecdysone peaks, necessary for pupariation. The regulation of steroid pulses by presence or absence of external nutrients is not known in vertebrates and is worth exploring further. Neuronal regulation of metabolic homeostasis from feeding to attaining satiety, is related to the internal state of the animal (Sternson, 2013). Steroid signaling has been studied extensively in two non-mammalian systems viz. *Drosophila* and *Arabidopsis* (Thummel and Chory, 2002). Additionally, steroid receptors regulate growth by inhibiting the Myc family of transcription factors in endocrine tissue (Delanoue et al., 2010). The similarity in these non-mammalian systems extends to mammalian systems in that here too steroid signaling is through nuclear receptors. However, though nuclear receptors have been implicated in nutrient sensing (Pardee et al., 2011), and steroid sensing mechanisms have been studied (Efeyan et al., 2015) the ability of neuronal nutrient sensing circuits to regulate steroid levels in mammals has not been tested.

The study of how nutrient deprivation and stress during development affects the health and physiology of the fully developed adult in a range of organisms is important because such studies will enable the understanding of common cellular signaling principles for nutrient sensing in a context dependent manner across evolution. *Drosophila* larvae provide an excellent opportunity for such studies, unlike mammals where development is intra-uterine. Consequently, experimental access to the developing organism is poor and if done, tends to affect the health of the mother, making the interpretation of these experimental results complex.

## Intracellular Calcium Signaling in Neurons and the Regulation of Metabolism in Multicellular Organisms

One way to identify possible unifying evolutionary principles underlying neural mechanisms that regulate nutrient sensing and developmental and/or behavioral outputs, is to investigate the role of signaling pathways that evolved in parallel with multicellularity. In this context, the intracellular calcium signaling pathway is of interest after it was identified as a key regulator of the neuronal response to protein deficiency in the third instar larval brain of *Drosophila* (Jayakumar et al., 2016). Evidence for genes encoding proteins like the inositol-1,4,5-trisphosphate receptor (IP_3_R), an intracellular calcium release channel; stromal interaction molecule (STIM), an endoplasmic reticular calcium sensor; Orai, a plasma-membrane localized calcium channel also referred to as the Store-operated calcium entry (SOCE channel); transient receptor channels (TRPs) that bring in extracellular calcium in response to a range of intracellular signals; as well as two-pore channels that mediate changes in levels of cytosolic calcium in response to extracellular stimuli, first appeared in the unicellular choanoflagellates, which precede multicellularity (Cai, 2008; Cai et al., 2014). Interestingly, ryanodine receptors (RyRs), another class of intracellular calcium release channels, are not found in choanoflagellates (Cai and Clapham, 2012). However, the calcium signaling toolkit has diversified vastly in multicellular organisms with several regulatory mechanisms for different physiological functions.

In vertebrates, IP_3_, generated by stimulation of either G-protein coupled receptors (GPCRs) or receptor tyrosine kinases (RTKs), binds to the IP_3_R on the endoplasmic reticulum and leads to elevated cytosolic calcium by calcium release from ER stores (Berridge, 1993; Berridge et al., 2000). Such release lowers ER store calcium leading to conformational changes in the ER membrane resident Ca^2+^ sensor STIM (Derler et al., 2016). Conformational changes in STIM allow multimerization and promote interaction with calcium channels on the plasma membrane known as Orai. Opening of Orai leads to influx of extracellular calcium into the cytosol, a process referred to as SOCE. The discovery of SOCE molecules, STIM and Orai and other molecules that regulate SOCE has been reviewed recently (Prakriya and Lewis, 2015). The calcium brought into the cytoplasm by SOCE is pumped back into the ER by an ER-localized Ca^2+^ pump (SERCA; Jousset et al., 2007). This mechanism of intracellular calcium signaling is conserved in invertebrates including *Drosophila* (Berridge et al., 2000; Cai et al., 2014). Though in vertebrate systems, RTKs also activate the IP_3_R (Berridge et al., 2000), genetic data do not support RTK activation of the IP_3_R in an invertebrate such as *Drosophila melanogaster* (Banerjee et al., 2006), suggesting that nutrient sensing mechanisms in multicellular organisms evolved through GPCRs. The fact that nutrient sensing in ancient fungi are GPCR dependent (Van Dijck, 2009) adds further credibility to such a hypothesis. Therefore, stimulus dependent cellular calcium signaling mechanisms could enable integration of nutrient loss with systemic metabolic regulation across phyla (Figure 1B).

Components of intracellular calcium signaling are essential in neurons for physiological functions ranging from cell and organismal viability to motor coordination (Knight et al., 2003; Joshi et al., 2004; Futatsugi et al., 2005; Venkiteswaran and Hasan, 2009; Subramanian et al., 2013b; Hartmann et al., 2014; Maus et al., 2017). It is known that mammalian receptors for the orexigenic neuropeptides NPY and ghrelin modulate intracellular calcium signaling (Minor et al., 2009; Racioppi and Means, 2012). Ghrelin is known to act through a G_q_-coupled receptor that further uses AMPK signaling (Racioppi and Means, 2012). However, the precise role of intracellular calcium signaling components like IP_3_R, STIM, Orai and SERCA in nutrient sensing and metabolic control have not been investigated directly, in part because vertebrates have multiple isoforms for all these molecules (Taylor and Tovey, 2010; Prakriya and Lewis, 2015). In *Drosophila* they are encoded by single genes (Cai, 2008; Prakriya and Lewis, 2015) allowing for genetic analysis of nutrient sensing by individual components of intracellular calcium signaling. Thus, the function of neuronal intracellular calcium signaling in the context of metabolic regulation needs to be investigated further in mammals where cellular mechanisms for nutrient sensing and regulation of appetite have been studied extensively (Sternson, 2013; Sternson and Eiselt, 2017).

There is also evidence demonstrating that intracellular calcium signaling is required in both neuronal and non-neuronal cells of *Drosophila*, for regulation of lipid metabolism (Baumbach et al., 2014a). *Drosophila*, mutants for the IP_3_R display hyperphagia and dysregulated lipid metabolism leading to obesity. These metabolic phenotypes could be rescued by restoration of IP_3_R function exclusively in peptidergic neurons (Subramanian et al., 2013a,b). Hyperphagia and subsequent resistance to starvation in the IP_3_R mutant flies suggest that intracellular calcium signaling in neurons regulates satiety. In *Drosophila* adipose tissues SOCE is a key regulator of fat storage, breakdown and related obesity (Baumbach et al., 2014a,b). Regulation of lipid metabolism by intracellular calcium signaling is also seen in mammalian muscle and liver tissue, where STIM and Orai regulate mitochondrial function for fatty acid oxidation and lipolysis (Futatsugi et al., 2005; Wang et al., 2012; Maus et al., 2017). Thus, intracellular calcium signaling may function in the regulation of metabolism in multiple cell types of metazoans. Regulation of feeding and metabolic phenotypes in both invertebrates and vertebrates by intracellular calcium signaling suggest a conservation of nutrient sensing mechanisms across phyla.

## Conclusions and Perspectives: The IP_3_R as an Integrator of Nutritional Inputs

The glutamatergic interneurons identified recently by us (Jayakumar et al., 2016), convey sensory signals received from cholinergic inputs to output peptidergic neurons. These glutamatergic neurons appear to function as integrators of multiple inputs that could include sensory and metabolic inputs from the fat-body (Koulakov et al., 2002). What makes these interneurons efficient integrators? One possibility is that calcium signaling through the IP_3_R allows such integration. The IP_3_R is thought to enable synaptic scaling. It has been speculated that the IP_3_R in mouse hippocampal and cerebellar Purkinje neurons is required for activity-dependent plasticity in dendrites (Berridge, 1993; Nakamura et al., 1999; Hartmann and Konnerth, 2005; Taylor and Tovey, 2010). The IP_3_R is also an established coincidence detector required for long term depression (LTD) in neuronal networks (Egger et al., 1999; Wang et al., 2000). Possibly, the IP_3_R plays a similar role in established mammalian neural circuits for regulating satiety. We propose that the neuronal IP_3_R allows integration of external nutrient information, obtained from various sensory modalities, with internal energy state and thus could influence metabolic set points that determine satiety. Modern day genetics in rodents affords experimental access that could address such questions directly in mammals (Nern et al., 2011; Taniguchi et al., 2011; Tsien, 2016). Results from such experiments would help answer whether intracellular calcium signaling in neurons affects the nutritional valence of the animal. It is possible that IP_3_R and intracellular calcium homeostasis allows flexible switching of hunger circuits to states that respond to different nutritional cues. A role for the IP_3_R in integrating nutrient availability with energy status of vertebrates might occur in the paraventricular nucleus of the hypothalamus (PVH) or in the insular cortex and needs to be investigated. It would also be interesting to test if neural components in the spinal cord regulate hypothalamic circuits given that spinal inputs are known to regulate the PVH (Geerling et al., 2010; Saper and Lowell, 2014). The identification of an mVG-mNSC axis in *Drosophila* that integrates nutrient availability with developmental check-points, suggests that it may be a precursor of a more evolved mammalian spinal cord-hypothalamic circuit.

Though the mechanism by which varying classes of nutrients are sensed differ among multicellular organisms, intracellular signaling cascades like IP_3_R signaling might allow context dependent modulation of nutrient sensing pathways. It could be speculated that the timing of ecdysteroid peaks during third instar larval progression are the result of integration of nutritional information and serve as additional checkpoints. The ecdysone receptor is thought to represent the insect equivalent of nuclear receptors that function in mammals for endocrine regulation of different physiological functions (Thornton, 2001; Pardee et al., 2011). Thus, IP_3_R mediated calcium release and associated SOCE in multicellular organisms might function to enable neuronal networks to appropriately adapt their physiological response to changes in their environmental conditions.

## Author Contributions

SJ researched and wrote the manuscript. GH critically revised the manuscript.

## Conflict of Interest Statement

The authors declare that the research was conducted in the absence of any commercial or financial relationships that could be construed as a potential conflict of interest. The reviewer TKH declared a shared affiliation, though no other collaboration, with one of the authors SJ to the handling Editor.
